# IGF-1 is not related to long-term outcome in hyperglycemic acute coronary syndrome patients

**DOI:** 10.1177/14791641211047436

**Published:** 2021-12-01

**Authors:** Cindya P Iswandi, Victor J van den Berg, Suat Simsek, Daan van Velzen, Edwin Ten Boekel, Jan-Hein Cornel, Sanneke de Boer, Maarten de Mulder, K Martijn Akkerhuis, Eric Boersma, Victor A Umans, Isabella Kardys

**Affiliations:** 1 Department of Cardiology, Erasmus MC, 6993 University Medical Center Rotterdam, Rotterdam, Netherlands; 2 Department of Cardiology, 1140 Northwest Clinics, Alkmaar, Netherlands; 3 Department of Internal Medicine, Northwest Clinics, Alkmaar, Netherlands; 4 Department of Clinical Chemistry, Northwest Clinics, Alkmaar, Netherlands

**Keywords:** Insulin-like growth factor-1, hyperglycemic, acute coronary syndrome, cardiovascular outcomes

## Abstract

**Purpose:**

Insulin-like growth factor-1 (IGF-1) has been associated with both protective and detrimental effects on the development of ischemic heart disease. The relationship between IGF-1 levels and major adverse cardiovascular events (MACE) in acute coronary syndrome (ACS) patients remains unclear. This study aimed to investigate the relationship between IGF-1 admission levels in hyperglycemic ACS patients and: (1) MACE over a 5 years follow-up, (2) type 2 diabetes at discharge, and (3) post-ACS myocardial infarct size and dysfunction.

**Methods:**

This was a post hoc analysis of the BIOMArCS-2 randomized controlled trial. From July 2008 to February 2012, 276 ACS patients with admission plasma glucose level between 140 and 288 mg/dL were included. Records of the composite of all-cause mortality and recurrent non-fatal myocardial infarction were obtained during 5 years follow-up. Venous blood samples were collected on admission. IGF-1 was measured batchwise after study completion. Oral glucose tolerance test was performed to diagnose type 2 diabetes, whereas infarct size and left ventricular function were assessed by myocardial perfusion scintigraphy (MPS) imaging, 6 weeks post-ACS.

**Results:**

Cumulative incidence of MACE was 24% at 5 years follow-up. IGF-1 was not independently associated with MACE (HR:1.00 (95%CI:0.99–1.00), *p* = 0.29). Seventy-eight patients (28%) had type 2 diabetes at discharge, and the highest quartile of IGF-1 levels was associated with the lowest incidence of diabetes (HR:0.40 (95%CI:0.17–0.95), *p* = 0.037). IGF-1 levels were not associated with post-ACS myocardial infarct size and dysfunction.

**Conclusions:**

IGF-1 carries potential for predicting type 2 diabetes, rather than long-term cardiovascular outcomes and post-ACS myocardial infarct size and dysfunction, in hyperglycemic ACS patients.

## Introduction

Regardless of intervention strategy, hyperglycemia is a known risk factor for future adverse clinical events in patients presenting with acute coronary syndrome (ACS).^1^ In a previous randomized controlled trial among hyperglycemic ACS patients, we compared intensive glucose control using intravenous insulin with conventional glucose management but did not find any differences in outcome, both during short ^2^ and long-term follow-up .^3^ Since insulin-like growth factor-1 (IGF-1) has been linked both to coronary artery disease (CAD) and glucose intolerance, investigating IGF-1 levels in patients that participated in this specific study could provide further pathophysiological insights.

Previous clinical studies have reported conflicting results about the role of IGF-1 in the pathophysiology of ischemic heart disease. Both protective and detrimental effects of IGF-1 have been associated with atherosclerotic processes leading to CAD .^4–9^ High levels of IGF-1 could on the one hand mediate the formation of atherosclerotic plaque by stimulating vascular smooth muscle cell proliferation and migration .^10^ On the other hand, IGF-1 has beneficial effects on cardiomyocyte function and survival by acting as a vascular protective factor and maintaining appropriate myocardial remodeling .^10,11^ The relationship between IGF-1 admission levels and long-term outcome in ACS patients, however, has not been extensively investigated .^12–14^ Particularly, the associations between IGF-1 admission levels and post-ACS myocardial remodeling remain unknown.

In addition to the role of IGF-1 in the pathophysiology of CAD, its role in glucose metabolism and homeostasis also deserves further attention .^15,16^ Previous studies have demonstrated that hyperglycemic patients have significantly lower levels of IGF-1 compared with normal subjects .^17,18^ As hyperglycemia on hospital admission has been linked to increased risk of subsequent cardiovascular mortality and morbidity among ACS patients,^1,17^ investigating the role of IGF-1 in hyperglycemic ACS patients might be of particular interest.

Therefore, the present study aimed to investigate the association of IGF-1 serum levels at hospital admission with mortality and recurrent non-fatal myocardial infarctions in hyperglycemic non-insulin dependent ACS patients who participated in the BIOMArCS-2 trial and were followed for 5 years. In addition, we investigated the association between IGF-1 admission levels and diabetes status at hospital discharge as well as post-ACS myocardial infarct size and dysfunction as measured by myocardial perfusion scintigraphy (MPS) at 6 weeks post-ACS.

## Materials and methods

### Study Population

The present study was a post hoc analysis of the “BIOMarker study to identify the Acute risk of a Coronary Syndrome 2” (BIOMArCS-2) glucose study. The design and primary results of the BIOMArCS-2 glucose study have been published previously .^2,3,19^ Briefly, in the BIOMArCS-2 glucose study, 280 hyperglycemic (admission plasma glucose level between 140 and 288 mg/dL) ACS patients were randomized to either 48 h of intensive glucose control using intravenous insulin or conventional glucose management, between July 2008 and February 2012. ACS was defined as typical ischemic chest pain with electrocardiographic (ECG) changes indicative of myocardial infarction (e.g. new ischemic ECG changes or elevated biomarkers of myocardial necrosis (creatine kinase, myocardial band [CK-MB] >16 U/L, or cardiac troponin I >0.45 ng/mL). Exclusion criteria were use of subcutaneous insulin, creatinine level >2.5 mg/dL, mechanical ventilation, or previously known left ventricular ejection fraction <30%. Consent was obtained from each patient after full explanation of the purpose and nature of all procedures used. The design of the BIOMArCS-2 glucose trial was approved by the Medical Ethics Committee Noord Holland and the trial was performed according to the principles of Declaration of Helsinki Version and in accordance with the Medical Research Involving Human Subjects Act. The trial is registered at www.trialregister.nl, with identifier NTR1205.

### IGF-1 measurements

IGF-1 measurement was performed in venous blood samples collected on admission for percutaneous coronary intervention in 276 BIOMArCS-2 patients. Serum IGF-1 levels were measured using an automated immunoanalyser iSYS (Immunodiagnostics (IDS), Germany) according to manufacturer’s instructions. The IDS-iSYS-IGF1 assay used is calibrated against the WHO international IGF-1 standard 02/254. The assay had a detection limit of 5 ng/mL and an inter assay coefficient of variation of 5%. The analyses were carried out at the ISO-15189 accredited Laboratory for Clinical Chemistry of Northwest Clinics Alkmaar.

### Endpoints

The primary endpoint of our study is the occurrence of major adverse cardiac events (MACE), defined as the composite endpoint of all-cause mortality and recurrent non-fatal myocardial infarction (MI). Follow-up lasted until January 2016. We obtained data on vital status from municipal registries, and data on MI by reviewing medical records. MI was defined as typical chest pain accompanied by new ischemic ECG changes on ECG or a rise and fall of troponins .^20^ Follow-up on all-cause death and MI was completed until January 2016 in 99.3% and 97.5% of patients, respectively. Patients with incomplete follow-up data were censored after their last hospital visit.

Secondary endpoints were the presence of type 2 diabetes at discharge and parameters of myocardial remodeling at 6 weeks post-ACS. During hospitalization, preferably on day three, an oral glucose tolerance test (OGTT) was performed accordingly in each patient. In line with the American Diabetes Association and WHO recommendations, a 2 h plasma glucose of ≥200 mg/dL or a fasting plasma glucose (FPG) of ≥126 mg/dL was defined as type 2 diabetes .^21,22^ Rest-gated MPS using technetium 99m-myoview single-photon emission computer tomography (SPECT) was used to investigate the infarct size and left ventricular function. Using commercially available software, polar maps were created of the relative distribution of tracer uptake throughout the entire left ventricle. Each polar map was normalized to its individual maximum and the defect size was defined as < 50% uptake area of the polar map and was subsequently expressed as a percentage of the left ventricle.^19^ The MPS-SPECT imaging was performed at 6 ± 1 week after the index event.

### Statistical analysis

Baseline demographic and clinical characteristics were compared among quartiles of IGF-1. Categorical data are described as numbers and percentages. Normally distributed continuous variables are presented as mean ± SD and non-normally distributed variables as median [Twenty fifth-Seventy fifth pecentile (IQR)]. Kolmogorov-Smirnov test was used to test the distribution of continuous data. Depending on distribution, ANOVA or Kruskal–Wallis test was applied to assess differences in continuous data. The χ^2^ or Fisher exact test when appropriate was used to study differences in categorical data.

A multivariable Cox proportional hazard model was used to investigate the association between continuous IGF-1 or IGF-1 quartiles and MACE. The model was adjusted for age, gender, and randomization for hyperglycemic therapy. To examine the relationship between IGF-1 and the presence of type 2 diabetes and the MPS-SPECT parameters, we used logistic regression models (diabetes) and linear regression models (MPS-SPECT), respectively. Statistical analyses were performed with IBM SPSS Statistics version 23 for Windows and a two-tailed *p* value <0.05 was defined as statistically significant.

## Results

The mean age of the patients in our study was 65 ± 11 years, 77.5% was male and 11% had previously had MI. The mean IGF-1 level at admission was 121.5 ± 36.3 ng/mL; IGF-1 level did not differ significantly between randomization groups; it was 120.8 ± 35.9 ng/mL for the intensive glucose management group vs 122.5 ± 36.9 ng/mL for the control group (*p* = 0.70). Table 1 presents the distribution of the clinical characteristics of the study population during hospital admission according to the quartiles of IGF-1. Patients in the lowest quartile of IGF-1 were older, more often female, and more likely to have hypertension than patients in higher IGF-1 quartiles.

**Table 1.** Patient characteristics, mean ± SD or *N* (%).

	First quartile (Q1) *N* = 68	Seconf quartile (Q2) *N* = 70	Third quartile (Q3) *N*= 69	Fourth quartile (Q4) *N* = 69	*p* Value
*IGF-1 (ng/mL)*	<94.5	94.5–118.4	118.5–145.4	>145.4	
Age (years)	70 ± 10	66 ± 11	62 ± 11	61 ± 12	0.001^*^
Male gender (%)	38 (56)	57 (81)	61 (88)	58 (84)	0.000^*^
BMI (kg/m^2^)	26.4 ± 3.4	26.4 ± 4.1	27.0 ± 3.3	26.4 ± 3.3	0.65
Waist (cm)	103 ± 12	101 ± 13	102 ± 9	100 ± 10	0.50
Current smoker (%)	27 (40)	28 (40)	27 (39)	23 (33)	0.99
Hypertension (%)	33 (49)	23 (33)	25 (36)	18 (26)	0.039^a^
Hypercholesterolemia (%)	16 (24)	19 (27)	20 (29)	16 (23)	0.84
Previous MI (%)	10 (15)	8 (11)	6 (9)	6 (9)	0.63
Previous DM (%)	11 (16)	6 (9)	6 (9)	4 (6)	0.19
STEMI at admission (%) Persistent (%) Non-persistent (%)	56 (82) 17 (25) 39 (57)	56 (80) 21 (30) 35 (50)	59 (86) 10 (14) 49 (71)	57 (83) 22 (32) 35 (51)	0.16
ACS management Invasive coronary angiography (%) Indication for CAG Elective (%) Emergency (%) Primary PCI (%) Fibrinolytic (%)	65 (95) 9 (13) 56 (82) 56 (82) 0 (0)	70 (100) 12 (17) 58 (83) 57 (81) 0 (0)	69 (100) 9 (13) 60 (87) 59 (86) 0 (0)	68 (99) 6 (9) 62 (90) 59 (86) 0 (0)	0.10 0.21 0.28 NA
Medications at discharge Aspirin (%) Clopidogrel (%) Beta-blocker (%) Statins (%) ACE-inhibitor (%) Angiotensin-2 receptor blocker (%)	63 (93) 52 (76) 64 (94) 65 (96) 46 (68) 10 (15)	68 (97) 50 (71) 69 (99) 68 (97) 50 (71) 6 (9)	67 (97) 43 (62) 68 (99) 68 (99) 54 (78) 5 (7)	67 (97) 53 (77) 68 (99) 68 (99) 52 (75) 7 (10)	0.99 0.20 0.99 0.59 0.73 0.43

^*^P<0.05.

BMI= body mass index, DM= diabetes mellitus IGF-1 = insulin-like growth factor-1, MI = myocardial infarction, STEMI = ST-elevation myocardial infarction.

### Long-term cardiovascular events

**Figure 1.**
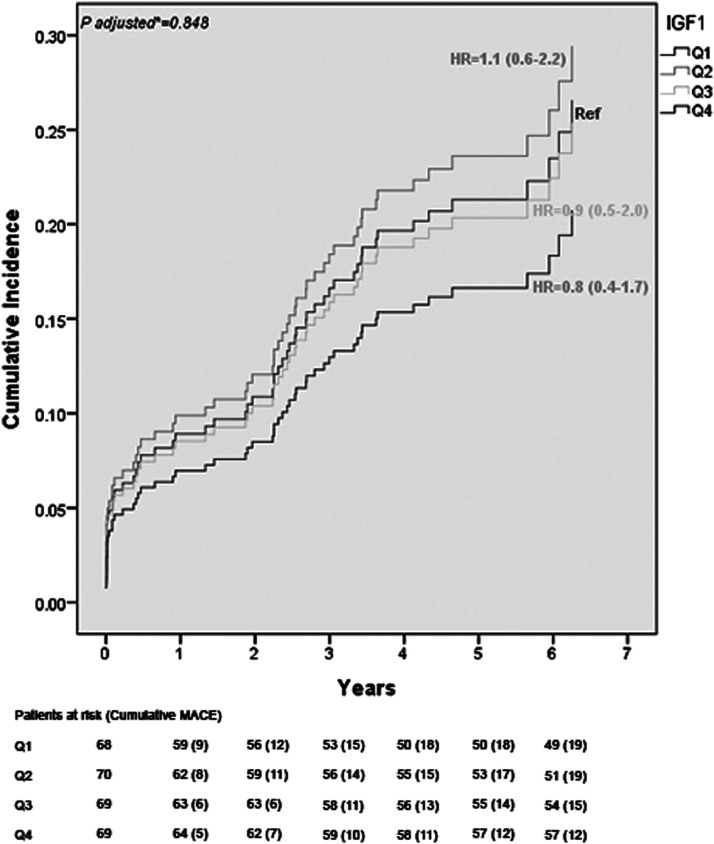
The probability of MACE, the composite of all-cause mortality, and non-fatal recurrent myocardial infarction, during a 5 years follow-up as a function of IGF-1 quartiles. The model was adjusted for age, gender, and randomization of hyperglycemic therapy. IGF-1 = insulin-like growth factor-1, MACE = major adverse cardiovascular events.

During a median follow-up of 5.1 years (IQR 4.0–6.2), 65 patients (24%) experienced a MACE. Of these 65 patients, 32 patients (49%) died from all causes. Patient in the highest quartile had a better chance of survival when compared to Q1 (Figure 1 and Table 2); however, particularly, after adjustments for age, gender, and randomization for hyperglycemic therapy, there was no longer any survival benefit. Continuous IGF-1 levels were not associated with MACE [adjusted hazard ratio (HR): 1.00 (95% CI 0.99–1.00)]. An overview of unadjusted and adjusted hazard ratio for MACE according to continuous levels of IGF-1 as well as IGF-1 quartiles, with the lowest quartile (Q1) as the reference is presented in Table 2.

**Table 2.** IGF-1 and the occurrence of MACE.

Parameters	MACE (*n*)	Univariable analysis		Multivariable analysis^a^	
		HR (95% CI)	*p* value	HR (95% CI)	*p* value
IGF-1 (ng/mL)	65	1.00 (0.99, 1.00)	0.29	1.00 (0.99, 1.00)	0.74
IGF-1 quartiles (ng/mL)					
Q1 (<94.5)	19	1.00 (ref)		1.00 (ref)	
Q2 (94.5–118.4)	19	0.92 (0.47, 1.83)	0.82	1.11 (0.55, 2.25)	0.78
Q3 (118.5–145.4)	15	0.77 (0.38, 1.56)	0.46	0.96 (0.45, 2.02)	0.90
Q4 (>145.4)	12	0.59 (0.28, 1.26)	0.18	0.78 (0.36, 1.72)	0.54

^**a**^Adjusted for age, randomization of hyperglycemic treatment, and gender.

HR= hazard ratio, IGF-1 = insulin-like growth factor-1, MACE = major adverse cardiovascular events (all-cause mortality and recurrent non-fatal myocardial infarction).

### Type 2 diabetes

At discharge, a total of 78 patients (28%) were classified with type 2 diabetes according to their OGTT. For each increase in IGF-1 quartile, the risk of diabetes at hospital discharge decreased (*p* for trend = 0.003). In a logistic regression model adjusted for age, gender, randomization of hyperglycemic therapy, and BMI, the odds ratios of having type 2 diabetes compared to the lowest quartile of IGF-1 were 0.48 (95% CI 0.21, 1.1) in Q2, 0.33 (95% CI 0.14, 0.81) in Q3, and 0.40 (95% CI 0.17, 0.95) in Q4 as presented in table 3.

**Table 3.** IGF-1 and type 2 diabetes at discharge.

Independent variable	Type 2 diabetes (*n*)	Univariable analysis		Multivariable analysis^a^	
		OR (95% CI)	*p* value	OR (95% CI)	*p* value
IGF-1 (ng/mL)	78	0.99 (0.98, 0.997)	0.008	0.99 (0.98, 1.00)	0.10
IGF-1 quartiles (ng/mL)					
Q1 (<94.5)	27	1.00 (ref)		1.00 (ref)	
Q2 (94.5–118.4)	20	0.43 (0.20, 0.92)	0.030	0.48 (0.21, 1.09)	0.079
Q3 (118.5–145.4)	15	0.32 (0.14, 0.70)	0.005	0.33 (0.14, 0.81)	0.016
Q4 (>145.4)	16	0.31 (0.141, 0.68)	0.003	0.40 (0.17, 0.95)	0.037

^*^Adjusted for age, randomization of hyperglycemic therapy, BMI, and gender in multivariate analysis.

*O*R= Odds ratio, IGF-1 = insulin-like growth factor-1.

### Infarct size

The median extent of myocardial infarct size was 3% [Interquartile range: 0%–11%] and the median left ventricular ejection fraction (LVEF) 6 weeks post-ACS was 59 [Interquartile range: 51–65]. We could not identify a statistically significant relationship between quartiles of IGF-1 and myocardial infarct size or LVEF (Table 4).

**Table 4.** Comparison of MPS-SPECT imaging parameters according to IGF-1 quartiles.

MPS-SPECT imaging parameters	Q1 (<94.5)	Q2 (94.5–118.4)	Q3 (118.5–145.4)	Q4 (>145.4)	*p* value^a^
LVEF	57 ± 12	57 ± 12	58 ± 9	57 ± 12	0.81
ESV	48 [30–79]	54 [35–70]	56 [38–72]	52 [39–78]	0.33
EDV	114 [85–150]	122 [98–153]	130 [110–157]	125 [110–147]	0.07
SRS	1.5 [0–11]	2 [0–8]	1 [0–7]	3 [0–8]	0.81
LV volume	81 [55–109]	86 [66–110]	93 [73–113]	88 [71–108]	0.14
Extent	2.5 [0–13]	3 [0–12]	1 [0–8]	4 [0.8–10]	0.91

^*^P Value of linear regression analysis between IGF-1 and MPS-SPECT parameters. Normally distributed continuous data are presented as mean ± SD, and skewed data as median [interquartile range]. EDV= end diastolic volume, ESV= end systolic volume, IGF-1 = insulin-like growth factor-1, LV = left ventricle, LVEF=left ventricular ejection fraction, SRS= summed rest score.

## Discussion

Previously, we have shown in the BIOMArCS-2 glucose study that intensive glucose management in hyperglycemic ACS patients does not lead to better outcomes than conventional treatment. Here, we demonstrate that: (1). IGF-1 admission levels of 276 non-insulin dependent hyperglycemic ACS patients were not associated with the composite of all-cause mortality and recurrent non-fatal MIs during a median of 5.1 years follow-up; (2). IGF-1 levels were significantly associated with type 2 diabetes at discharge with an increased risk in the lowest quartile; (3). IGF-1 levels were not independently associated with myocardial infarct size and dysfunction as assessed by MPS-SPECT imaging parameters at 6 weeks follow-up post-ACS.

To the best of our knowledge, the present study investigating the relationship between IGF-1 levels in ACS patients and clinical outcome is the one with the longest follow-up to date. In addition, we are the first to investigate the relationship between IGF-1 levels and infarct size as evaluated using MPS-SPECT. We show that IGF-1 at hospital admission of non-insulin dependent hyperglycemic ACS patients fails to predict long-term cardiovascular outcomes over the 5 years follow-up. The results are in concordance with the study by Wallander et al. among 575 diabetic patients with suspected ACS, which found that IGF-1 levels at hospital admission, discharge, 3 months, and 12 months after the index event were not related to cardiovascular death over a 3 years follow-up .^23^ Conversely, in a study by Bourron et al. among 1005 patients with ACS, low levels of age-adjusted IGF-1 at hospital admission were associated with worse cardiovascular outcomes over a 2 years follow-up, especially among diabetic patients .^14^ In contrast to our study, in both the study by Wallander et al. and the study by Bourron et al., ACS management was not performed according to currently prevailing guidelines. In the DIGAMI-2 study, only little under a half of the included patients underwent acute revascularization and double-antiplatelet therapy was provided in approximately 20% of the patients ,^23^ while in the study of Bourron PCI was performed in approximately 65% .^14^

Overall, the discrepancies between the studies are probably caused by differences in study populations. Patients with CAD and hyperglycemia are known to have lower IGF-1 levels .^6,7,17,18^ Therefore, our study population of hyperglycemic ACS patients can be expected to have relatively low baseline IGF-1 levels. Indeed, IGF-1 levels within our cohort ranged between less than 94.5 ng/mL in Q1 and higher than 145 ng/mL in Q4. These levels are substantially lower compared to the age-adjusted IGF-1 levels within the cohort investigated by Bourron, which ranged between less than 135 ng/mL in Q1 and higher than 283 ng/mL in Q4, although the study was also performed in ACS patients .^14^ Our data thus confirms the results of previous studies and extends them to current age ACS-treatment, indicating that ACS patients with hyperglycemia have lower levels of IGF-1, regardless of intervention strategy .^17^

We found that the lowest quartile of IGF-1 concentration is associated with an increased risk of type 2 diabetes at discharge among hyperglycemic ACS patients. Our results support the study by Wallander et al. among 168 ACS patients which found that low IGF-1 levels may be a useful predictor of abnormal glucose metabolism in ACS patients .^17^ In line with our study, Teppala et al. also found that low levels of IGF-1 are associated with type 2 diabetes in a population aged less than 65 years; but here, the association disappeared in those aged ≥65 years .^18^ However, our findings showed that low levels of IGF-1 are associated with the presence of type 2 diabetes among ACS patients with an interquartile range of age between 56 and 72 years. These results might provide additional evidence that further supports the association between IGF-1 and diabetes, regardless of age .^16^ Physiological mechanisms underlying the role of IGF-1 in glucose metabolism remain unclear, yet an association between low IGF-1 levels and diminished hepatic insulin sensitivity has been observed .^10^

Post infarction left ventricular (LV) dysfunction is characterized by progressive LV dilatation and hypertrophy as well as wall thinning in the infarcted tissue .^24,25^ Progressive LV dysfunction and larger infarct size are associated with poor prognosis in ACS patients .^25^ We found that IGF-1 does not independently predict myocardial infarct size and LV dysfunction. In contrast, a small study among 34 ACS patients by Lee et al. showed that higher IGF-1 levels are associated with higher LVEF and smaller left ventricular dimensions, as assessed by echocardiography at 1 week post-ACS .^26^ These differences in results could be caused by differences in study population, as we only included hyperglycemic patients with relatively low IGF-1 average levels (121.5 ± 36.2 ng/mL in our study vs 280.8 ± 35.3 ng/mL in Lee et al. ^26^), the larger sample size in the present study which reduces the probability of a chance finding, and the diagnostic tools used to evaluate myocardial function. Previous studies have shown that MPS-SPECT imaging is superior to echocardiography for examining LV function and myocardial viability because MPS-SPECT imaging identifies viable and infarcted myocardium based on regional differences in radiotracer uptake .^25,27,28^

### Study limitations

This investigation being part of the BIOMArCS-2 study, participants were limited to ACS patients with non-insulin dependent hyperglycemia. The overall number of MACE in the study was modest (*n* = 65), and due to the small number of events within the individual IGF-1 quartiles, statistical power to detect differences between groups may have been limited. Another limitation of this study is that the presence of type 2 diabetes was assessed prior to hospital discharge, but not confirmed afterward. Previous studies have shown that disturbances in glucose metabolism diagnosed by OGTT before hospital discharge in ACS patients might represent a temporary finding rather than latent pre-existing diabetes .^29,30^ Nevertheless, our findings may be deemed robust because recent studies have shown that OGTT is a sensitive method, especially compared with admission plasma glucose or FPG, to investigate the presence of previously undiagnosed diabetes in hyperglycemic patients with ACS and to give reliable information about long-term state of glucose metabolism .^31–35^ The choice of performing post-ACS imaging at 6 weeks follow-up might have excluded patients with the largest infarctions, who are more likely to die early, and therefore may have limited the ability to detect associations of IGF-1 with LV perfusion defect size.

## Conclusions

In conclusion, IGF-1 serum levels at hospital admission in hyperglycemic non-insulin dependent ACS patients are not associated with increased risk of subsequent cardiovascular mortality and morbidity over a 5 years follow-up and fail to predict myocardial infarct size and dysfunction as measured by MPS-SPECT. However, the lowest quartile of IGF-1 concentration is associated with an increased risk of type 2 diabetes. Altogether, these results suggest that IGF-1 could be more useful for predicting type 2 diabetes than long-term cardiovascular outcomes in hyperglycemic ACS patients.
